# A fast direct solver for surface-based whole-head modeling of transcranial magnetic stimulation

**DOI:** 10.21203/rs.3.rs-3079433/v1

**Published:** 2023-07-10

**Authors:** S. N. Makaroff, Z. Qi, M. Rachh, W. A. Wartman, K. Weise, G. M. Noetscher, M. Daneshzand, Zhi-De Deng, L. Greengard, A. R. Nummenmaa

**Affiliations:** 1 Electrical and Computer Engineering Department, Worcester Polytechnic Institute, Worcester, MA 01609 USA; 2 Athinoula A. Martinos Ctr. for Biomedical Imaging, Massachusetts General Hospital, Harvard Medical School, Charlestown, MA 02129 USA; 3 Center for Computational Mathematics, Flatiron Institute, New York, NY 10010 USA; 4 Max Planck Institute for Human Cognitive and Brain Sciences, Stephanstr. 1a, 04103, Leipzig Germany; 5 Technische Universität Ilmenau, Advanced Electromagnetics Group, Helmholtzplatz 2, 98693 Ilmenau Germany; 6 Computational Neurostimulation Research Program, Noninvasive Neuromodulation Unit, Experimental Therapeutics & Pathophysiology Branch, National Institute of Mental Health, NIH 10 Center Drive, Bethesda, MD 20892 USA; 7 Courant Institute of Mathematical Sciences, 251 Mercer Street, New York, NY 10012 USA

**Keywords:** Transcranial Magnetic Stimulation, Numerical Modeling, Direct Near Real Time TMS Solution, Boundary Element Fast Multipole Method BEM-FMM, FMM Inverse, FMM-LU, Fast TMS Motor Mapping, Precise TMS Motor Mapping, Biophysical Modeling

## Abstract

**Background:**

When modeling transcranial magnetic stimulation (TMS) in the brain, a fast and accurate electric field solver can support interactive neuronavigation tasks as well as comprehensive biophysical modeling.

**Objective:**

We formulate, test, and disseminate a direct (*i.e.,* non-iterative) TMS solver that can accurately determine global TMS fields for any coil type everywhere in a high-resolution MRI-based surface model with ~200,000 or more arbitrarily selected observation points within approximately 5 sec, with the solution time itself of 3 sec.

**Method:**

The solver is based on the boundary element fast multipole method (BEM-FMM), which incorporates the latest mathematical advancement in the theory of fast multipole methods – an FMM-based LU decomposition. This decomposition is specific to the head model and needs to be computed only once per subject. Moreover, the solver offers unlimited spatial numerical resolution.

**Results:**

Despite the fast execution times, the present direct solution is numerically accurate for the default model resolution. In contrast, the widely used brain modeling software SimNIBS employs a first-order finite element method that necessitates additional mesh refinement, resulting in increased computational cost. However, excellent agreement between the two methods is observed for various practical test cases following mesh refinement, including a biophysical modeling task.

**Conclusion:**

The method can be readily applied to a wide range of TMS analyses involving multiple coil positions and orientations, including image-guided neuronavigation. It can even accommodate continuous variations in coil geometry, such as flexible H-type TMS coils. The FMM-LU direct solver is freely available to academic users.

## 1. Introduction

Transcranial magnetic stimulation (TMS) is a non-invasive technique used to stimulate specific regions of the brain. For TMS to be a more effective and personalized therapeutic tool, it is crucial to accurately simulate the induced electric field (E-field) distribution in the brain. For interactive E-field based TMS neuronavigation, the E-field should be updated and displayed fast enough to provide the operator with a sense of real-time interaction.

There are several time scales to consider. First, most real-time visualization applications have a minimum update rate of around 15–30 frames per second (fps) to have a smooth and continuous experience; this allows for approximately 30–60 ms for coil position update, E-field solution update, and image rendering. The second time scale of relevance relates to the pace at which a human or a robot [1],[2] could move a TMS coil from one position to another. Given an estimate for sequential robotic targeting speed of ~1cm/s [1], and if we consider coil position change of a few millimeters to produce a meaningful change in the E-field, then the solution can be updated on the time scale on the order of 100 ms. The final time scale is that of the TMS interstimulus interval, if the E-field solution were to update synchronous with TMS pulse delivery. For conventional motor mapping with single pulses, an approximately ~5 s interstimulus interval is typical to avoid inhibitory effects (cf. [3],[4]).

Over the past two years, significant progress has been made with regard to excellent real-time high-resolution solvers for TMS field modeling [5],[6],[7], which execute in approximately 20–50 ms. These simulation speeds are close to the standard screen refresh rate and should be very useful in practice for continuous smooth field visualization. In general, these solvers do not involve accelerating the main numerical algorithm itself but rather apply highly efficient and “smart” interpolation methods to thousands of precomputed solution sets.

This study aims to introduce a direct accurate TMS solver that can output TMS fields from any coil and anywhere in a high-resolution head model within ~3–5 sec. This solver is based on the boundary element fast multipole method or BEM-FMM [8],[9],[10],[11] and the most recent mathematical advance in the theory of fast multipole methods – an FMM-based LU factorization or FMM-LU [12]. It does not use any interpolation routines or precomputed solutions, but does require initial LU decomposition of a head-model matrix, which takes approximately 40 min.

One motivation for this study is a modern precise motor mapping protocol [13], which would benefit from for fast accurate field calculations while the participant is still in the hospital or facility so that a follow up TMS session could be avoided. In addition, the mapping process could be further optimized through adaptive algorithms that will utilize a sequential approach for estimating the “hot spot” [14]. On the other hand, the fast solver can be readily utilized to precompute a large number of E-field solutions covering “all possible coil positions/orientations” of interest that can be subsequently used to stimulate a desired cortical target derived from anatomical of functional connectivity data (see, *e.g.,* [15]). The precalculated solutions could also be used to guide the operator to the right target in an interactive neuronavigation setting.

In contrast to the finite element method, BEM formulations are restricted to the surface of compartment domains, thereby reducing the dimensionality of the problem for brain modeling in general. There has been a resurgence of interest in these surface-based methods due to the availability of fast algorithms such as fast multipole methods (FMMs) [16],[17]. Assuming *N* is the number of degrees of freedom used in sampling surfaces and *A* is the *N* × *N* system matrix (*Ax* = *b*) obtained after the application of a suitable quadrature rule to an integral representation, these algorithms permit *A* to be applied to a vector in *O*(*N*) or *O*(*N logN*) time. For well-conditioned systems, this allows for the rapid iterative solution of very large-scale problems. The recently developed charge-based boundary element fast multipole method (BEM-FMM) [8],[9],[10],[11] utilizes this type of iterative solution. It requires 20–50 iterations on average and executes in 30–60 s depending on the required accuracy. These data are for high-resolution surface-based head models such as those generated by FreeSurfer [19],[20] and SPM/CAT [21].

There is an obvious task where iterative solvers are not satisfactory. This includes near real-time E-field predictions for TMS brain mapping or stimulation planning with a large number of possible coil positions, orientations, or even coil geometry changes. Each such setup gives rise to a unique right-hand side. Here, direct solvers would be preferred to solve the same system matrix with *multiple* right-hand sides. Along with this, fast direct solvers could be useful when exploring low-rank perturbations of the head geometry (and/or tissue conductivities) and, hence, the system matrix. Updating the solution in such cases requires only a few applications of *A*^−1^ or a fast update of the inverse itself [22],[23].

In the last few years, several algorithmic ideas have emerged which permit the construction of a compressed approximation of *A*^−1^ at a cost of the order *O*(*N*) or *O*(*Nlog*_*p*_*N*), for modest *p*. To construct the first fast direct TMS solver, we apply one such scheme [12], which is referred to as the FMM-LU method [12]. It uses FMM-type hierarchical compression strategies to rapidly compute an LU-factorization of the large system matrix.

## 2. Materials and Methods

### 2.1. Charge-based BEM in the succinct form

Induced electric charges with a surface charge density *ρ*(***r***) will reside on every tissue conductivity interface *S* once an external electromagnetic stimulus or a primary E-field ***E***^*p*^(***r***) of a TMS coil is applied. These induced surface charges will alter the primary field to fulfill the law of current conservation across the boundaries. The E-field generated by all surface charges anywhere in space except the charged interfaces themselves is governed by Coulomb’s law. The total E-field ***E***(***r***) becomes the sum of the primary field and the secondary charge field i.e.,

(1) $$\text{E}(\text{r})={\text{E}}^{p}(r)+\frac{1}{4\pi \varepsilon_{0}}\int_{S}\frac{r-r^{\prime }}{{\left|r-r^{\prime }\right|}^{3}}\rho \left(r^{\prime }\right)dr^{\prime },\;r\notin S$$

where *ε*_0_ is dielectric permittivity of vacuum (a normalization constant). The E-field in Eq. (1) is discontinuous at the interfaces. When approaching a charged interface *S* with a certain normal vector ***n*** and assigning index *into* the medium from which ***n*** is pointing and index *out* of the medium toward which ***n*** is pointing, the E-field close to the boundary is given by two limiting values [29]

(2) $${\text{E}}_{\text{in/out}\;}(r)={\text{E}}^{p}(r)+\frac{1}{4\pi \varepsilon_{0}}\int_{S}\frac{r-r^{\prime }}{{\left|r-r^{\prime }\right|}^{3}}\rho \left(r^{\prime }\right)dr^{\prime }\mp \text{n}(r)\frac{\rho (r)}{2\varepsilon_{0}},\;r\in S$$

where ${\text{E}}_{\text{in/out}\;}(r)\equiv \underset{\Delta \to 0}{\lim}\text{E}(r\mp \Delta \text{n}(r))\text{ and }r\mp \Delta \text{n}(\text{r})$ and ***r*** ∓ Δ***n***(***r***) is not on the surface. The second term on the right-hand side of Eq. (2) is a continuous contribution of all other surface charges while the last term is a discontinuous contribution of a local planar sheet of charge located exactly at ***r*** resulting in a jump of the normal E-field by *ρ*(***r***)/*ε*_0_. The “discrete” interpretation is that the integral on the right-hand side of Eq. (2) is the continuous contribution of surface charges of all facets except the facet located exactly at ***r*** while the last term on its right-hand side is a discontinuous contribution of the facet located exactly at ***r***. This facet is a planar sheet of charge.

An integral equation for *ρ*(***r***) is obtained after substitution of Eq. (2) into the quasistatic boundary condition which enforces the continuity of the normal component of the electric current across the interface. That is,

(3) $$\sigma_{\text{in}\;}\text{n}(r)\cdot {\text{E}}_{\text{in}\;}(r)=\sigma_{\text{out}\;}\text{n}(r)\cdot {\text{E}}_{\text{out}\;}(r),\;r\in S$$

Here, *σ*_*in*_, *σ*_*out*_ are the conductivities just inside and outside with respect to the direction of the normal vector. After combining similar terms, a Fredholm equation of the second kind is obtained

(4) $$\frac{\rho (r)}{2}-K(r)n(r)\cdot \int_{S}\frac{1}{4\pi }\frac{r-r^{\prime }}{{\left|r-r^{\prime }\right|}^{3}}\rho \left(r^{\prime }\right)dr^{\prime }=K(r)n(r)\cdot E^{p}(r),\;r\in S$$

where *K* is the electric conductivity contrast $K=\frac{\sigma_{\text{in}\;}-\sigma_{\text{out}\;}}{\sigma_{\text{in}\;}+\sigma_{\text{out}\;}}.$.

### 2.2. Iterative (BEM-FMM) versus direct (FMM LU) solution of Eq. (4)

Assuming that the charge density *ρ*(***r***) has a constant value *x*_*m*_ for every triangular facet *t*_*m*_ with area *A*_*m*_, Eq. (4) is discretized in standard matrix form ($E_{m}^{p}$ is the primary field at the *m*-th facet)

(5) $$Ax=b,\;A_{mn}=\frac{1}{2}\delta_{mn}-\frac{K}{A_{m}}n_{m}\cdot \iint_{t_{m}t_{n}}\frac{1}{4\pi }\frac{\left(r-r^{\prime }\right)}{{\left|r-r^{\prime }\right|}^{3}}dr^{\prime }dr,b_{m}=\frac{K}{A_{m}}n_{m}\cdot E_{m}^{p}$$

where *δ*_*mn*_ is Kronecker delta. Matrix *A* is well conditioned. Therefore, an iterative solution of Eq. (5) with the generalized minimum residual method (GMRES) [18] converges fast, in 20–40 iterations. FMM is used to compute the matrix-vector product in a “matrix free” fashion so that matrix *A* is never computed/stored [30],[26]. This solution was used previously [8],[9],[10],[11].

Another way to solve Eq. (5) is to apply FMM to a direct LU-decomposition of matrix *A*. In other words, a compressed approximation of *A*^−1^ is found directly so that a direct solution *x* = *A*^−1^*b* is attempted, without using an iterative method and for multiple right-hand sides, *b*. While matrix *A* is determined by the head model, the right-hand sides describe coil fields. This is the compression algorithm of Ref. [12], which is applied in this study. Indeed, the compressed inverse differs from the iterative solution. We will show that this difference is vanishingly small.

### 2.3. Human models and coil models used to test the algorithm

First, we test the default example of FEM software SimNIBS v3.2.6 [25] – the Ernie head model (~850,000 facets) with the default Magstim 70 mm coil model targeting the *M*1_HAND_ area of the left hemisphere and thoroughly described in [24]. The MRI segmentation was done with the headreco pipeline [24], which uses the SPM12/CAT [31] toolbox.

Second, we test four Connectome Young Adult [33] subjects and target the *M*1_HAND_ area of the left hemisphere with the MRiB91 coil of MagVenture using the sulcus-aligned coil positioning [32]. All head models (~830,000 facets each) have been obtained via FreeSurfer segmentation [19],[20]. The coil is approximated by ~30,000-elementary current elements (with the skin effect included) and the coil fields are also computed via the fast multipole method [34].

Finally, we test a healthy subject scanned at Max Planck Inst. for Human Cong. & Brain Sciences Leipzig, Germany with the MagVenture CB65 coil and the same left *M*1_HAND_ target. However, this model is manually refined in the region of interest (ROI) so that its overall size is 1,8 M facets. In all cases, the default SimNIBS conductivity parameters [24] have been used.

### 2.4. Testing method

Both accuracy and speed are tested. Two error types are considered: the relative error in the vector E-field, *Error*_*total*_, and the error in the magnitude of the E-field, *Error*_*mag*_

(6) $$\;\text{Erro}r_{\text{tota}l\;}=\frac{∥E_{t}(r)-E_{t}^{\prime }(r)∥}{∥E_{t}(r)∥},\;\;\text{Erro}r_{\text{mag}\;}=\frac{∥\left|E_{t}(r)\right|-\left|E_{t}^{\prime }(r)\right|∥}{∥\left|E_{t}(r)\right|∥}$$

over an arbitrary domain of interest where ∥·∥ is a 2-norm for a vector or scalar field. Index *t* denotes the total E-field in the head which is a sum of the primary field and the secondary field. As a ground truth, an iterative BEM-FMM solution with 54 million facets (a 1:64 uniform mesh refinement of all head compartments for the Ernie model), the FMM precision [26] of 1e-6, and with a terminal residual of 1e-6 has been utilized.

## 3. Results

### 3.1. Method accuracy versus SimNIBS FEM accuracy

For the default Ernie model example [24] of the FEM SimNIBS software [25], Table 1 gives the field error values for a mid-surface between the gray and white matter with ~200,000 observation nodes. Three solutions are considered: the default SimNIBS FEM solution, the iterative BEM-FMM solution, and the direct FMM LU solution – the subject of this study. For the iterative BEM-FMM solution, we also provide results for the uniformly refined models.

The iterative BEM-FMM solution in Table 1 uses the FMM precision of 1e-4 and the terminal residual of 1e-6. The FMM LU uses the same FMM precision of 1e-4. Two measures of error are given: one is for the entire midsurface between gray and white matter; the second covers a region of interest (ROI) on the midsurface contained within a 4 cm diameter sphere at the target point on the coil axis crossing the gray matter interface.

### 3.2. Method accuracy at different levels of internal FMM precision

Table 2 gives the field error values for the mid-surface between gray and white matter. Two solutions are compared against each other: the iterative BEM-FMM solution and the BEM-FMM LU solution. The iterative BEM-FMM uses the FMM precision of 1e-4 everywhere, including the field computations, and the terminal residual of 1e-6. It is considered as the ground truth. The FMM LU is tested with different values of FMM accuracy for (i) decomposition; and (ii) field computations. Four Connectome Young Adult models, subjects 101309, 110411, 117122, 120111 [28], are tested when the MRiB91 coil of MagVenture is targeting the *M*1_HAND_ of the left hemisphere. Along with this, the Ernie model provided with SimNIBS FEM software, segmented with the standard headreco settings, and the default coil type/position is also tested. The superscript denotes standard deviation.

### 3.3. Method speed

**Fig. 1.**
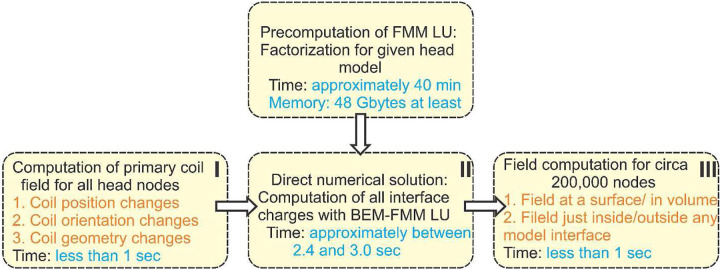
Approximate execution times of the method when the parameters from Table 2 are used for the FMM-LU solution (the Ernie model). The data are averaged for four workstations with Intel(R) Xeon(R) Gold 6348 CPU @ 2.60GHz 512 GB RAM, 56 cores, OS Windows Server 2022 Standard; MATLAB 2021b platform. The entire solution executes in slightly less than 5 sec.

The method speed was tested using four nearly identical workstations with Intel(R) Xeon(R) Gold 6348 CPU @ 2.60GHz, 512 GB RAM, 56 cores, OS Windows Server 2022 Standard and the common MATLAB 2021b platform. Fig. 1 outlines the corresponding execution times including precomputations. In Fig. 1, initial computations of the primary coil field and the final field computations for the given domain (steps I and III) take less than 1 sec for the FMM precision levels 1e-1 and 1e-2 while the direct numerical solution (step II) takes 2.4 s for the FMM precision level 1e-3 and 3.0 s for the FMM precision level 1e-4, respectively.

Equivalent SimNIBS v3.2.6/4.0 execution times on high-performance workstations were approximately 40 s. SimNIBS run times on a 2.3 GHz laptop are faster – approximately 30 s.

### 3.4. Example #1. Cortical and subcortical fields computed in 4.7 sec (MRiB91 coil)

**Fig. 2.**
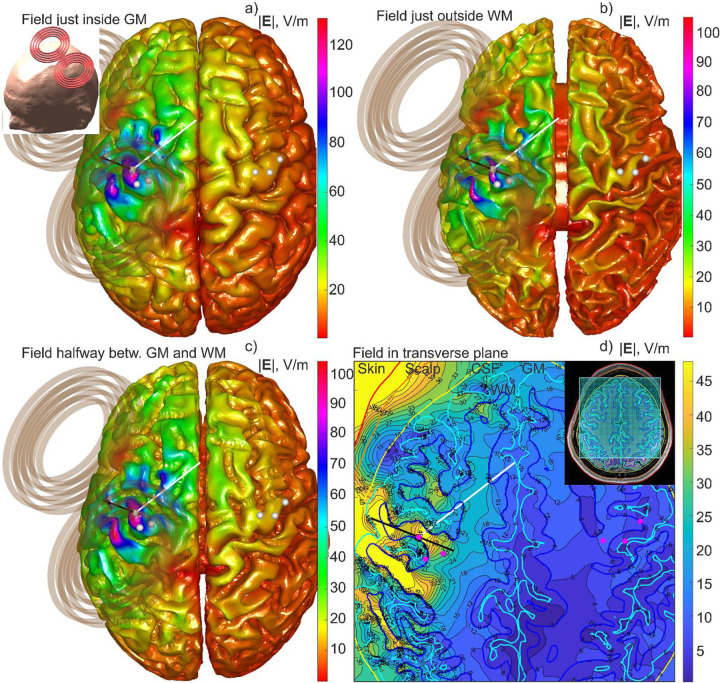
Different forms of the simulation output for Connectome subject 120111: a) Total E-field just inside the gray matter interface; b) Total E-field just outside the white matter interface; c) Total E-field at the cortical midsurface; d) Total E-field in a transverse plane beneath the coil. The entire computational sequence for any type of the output runs in approximately 4.7 s including graphical rendering in MATLAB.

As the first computational example, Fig. 2 shows the simulation output for Connectome Young Adult subject 120111 including: a) the total E-field just inside the gray matter interface; b) the total E-field just outside the white matter interface; c) the total E-field at the cortical midsurface and; d) the total E-field in a transverse plane beneath the coil. The MRiB91 coil of MagVenture is driven with *dI*/*dt* = 9.4*e*7 A/s. The computational sequence for any type of output from Fig. 2 takes approximately 4.7 seconds to run, including graphical rendering in MATLAB.

### 3.5. Example #2. Cortical and subcortical fields computed in 4.8 sec (authentic H1 coil)

**Fig. 3.**
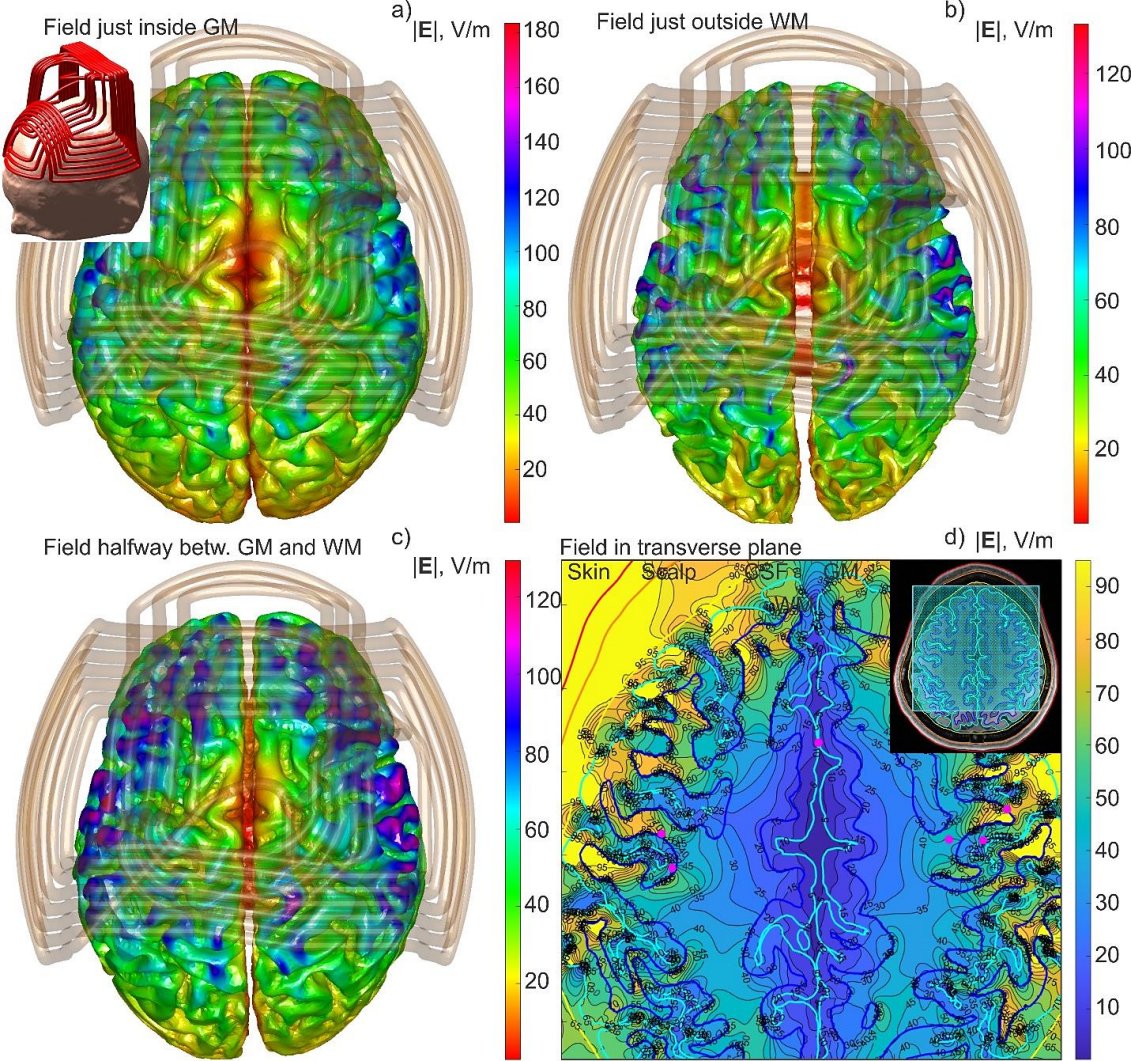
Different forms of the simulation output for Connectome subject 120111 with the H1 flexible coil of BrainsWay, Ltd: a) Total E-field just inside the gray matter interface; b) Total E-field just outside the white matter interface; c) Total E-field at the cortical midsurface; d) Total E-field in a transverse plane beneath the coil. The entire computational sequence for any type of the output runs in approximately 4.8 s including graphical rendering in MATLAB.

As a second example, Fig. 3 shows a simulation output for the same Connectome subject 120111 but when an H1 coil of BrainsWay is used. In this case, the coil is made *flexible*, and it changes its shape when continuously aligned with the patient’s head. Therefore, step I in Fig. 1 cannot be replaced by an interpolation of the precomputed coil field. The computational sequence for any type of output from Fig. 3 takes approximately 4.8 seconds to run, including graphical rendering in MATLAB.

## 4. Discussion

### 4.1 Iterative and direct FMM solutions produce nearly identical results

Table 2 indicates that the accuracy of FMM LU is *non-distinguishable* from the accuracy of BEM-FMM given the FMM precision level of 1e-4 or better used for constructing the compressed inverse. In this case, the factorization data stored in MATLAB workspace have the size of approximately 50 Gigabytes; their creation requires approximately 70 min. The direct solution – step II in Fig. 1 – executes in 3.0 s. The FMM precision level used for the field computations – steps I and III in Fig. 1 – may be as low as 1e-1 or 1e-2 without deteriorating the solution accuracy.

When the FMM precision level is reduced to 1e-3, the factorization data will have the size of 30 Gigabytes, their generation requires approximately 40 min, and the direct solution (step II in Fig. 1) executes in 2.4 s. This option could likely be preferred since its accuracy reported in the last four rows of Table 2 is approximately 0.4% on average for the critical ROI fields.

As Table 2 demonstrates, the FMM precision level used for the field computations has little influence on the solution accuracy. This is because in both BEM-FMM and FMM LU, the near-field interaction integrals are computed analytically and then substituted into the FMM pipelines. On the other hand, this level has a substantial influence on the method speed. Therefore, the values of 1e-2 or 1e-1 are preferred.

### 4.2 Solid agreement with SimNIBS is achieved for a refined head model

**Fig. 4.**
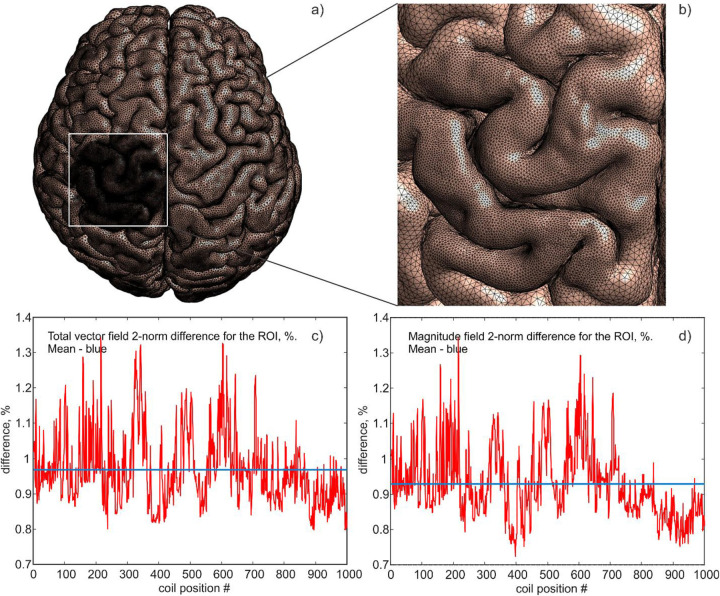
Default headreco segmentation of the healthy subject in a) was manually refined for cerebrospinal fluid, gray matter, and white matter within the ROI in a,b). One thousand different coil positions have been tested while scanning the ROI; SimNIBS and BEM-FMM solutions were compared with each other in c,d).

The lower 1^st^-order FEM accuracy from Table 1 can be improved using selective mesh refinement in the ROI domain as illustrated in Fig. 4a,b. Here, the default headreco segmentation [24] of the healthy subject was manually refined for cerebrospinal fluid, gray matter, and white matter (both surface and volume meshes were refined) as shown in Fig. 4b. One thousand different coil positions (using a MagVenture CB65 coil) have been tested while scanning the ROI.

Solutions using the refined meshes from SimNIBS and BEM-FMM were compared with each other as shown in Fig. 4c,d, respectively. The average vector field and magnitude field differences from (Eq. 6) within the ROI in Fig. 4c,d now attain the *sub-percent* values, which confirms the convergence of both methods to the same result. Due to the differences in the 2D *vs*. 3D numerical formulations of the problems for the BEM-FMM and SIMNIBS, respectively, some discrepancies remain close to the conductivity boundaries where the normal electric field is discontinuous. The practical implications of these differences require further investigation.

### 4.3. Solid agreement with SimNIBS is achieved for activating thresholds of intracortical neural cells for a refined head model

**Fig. 5.**
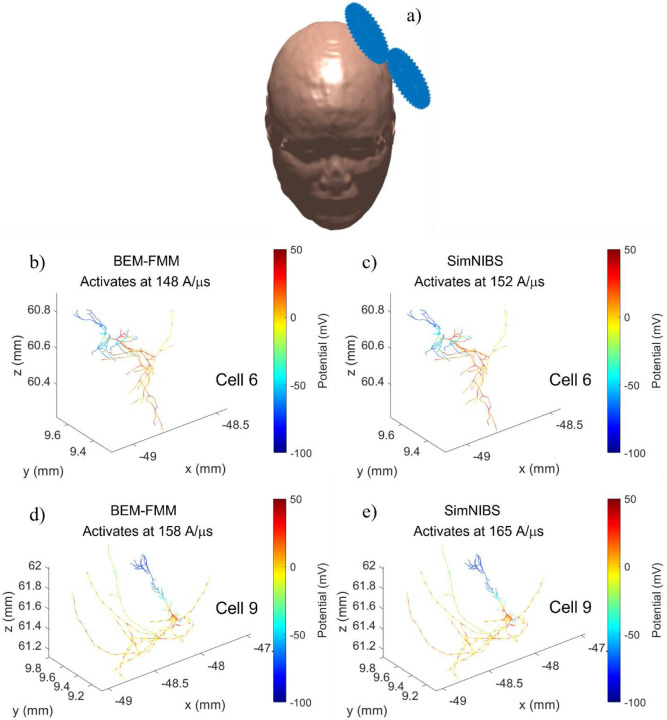
a) Coil position for the Ernie model. b)-e) activating thresholds and action potentials at 0.7 ms after activation.

Three intracortical neural cells (#4, 6, and 9) from a multi-scale toolbox Neuron Modeling for TMS (NeMo-TMS) [37] were placed 1 mm below the grey matter surface of the refined Ernie model (0.4 nodes per mm^2^) on the coil axis as shown in Fig. 5a and their activating thresholds were computed as a function of the E-field intensity, *dI*/*dt*, of a MagStim 70 mm coil (the SimNIBS coil model is used) for the default NeMo-TMS pulse form. Fig. 5b)-e) below compares the activating thresholds computed with BEM-FMM and SimNIBS, respectively, using the corresponding quasi-potentials. It also illustrates the action potentials at the neuron activation for cells 6 and 9, respectively, at 0.7 ms. The activating thresholds differ by 3.2% (cell 4), 2.6% (cell 6), and 4.2% (cell 9) with BEM-FMM always predicting slightly lower threshold values while the action potentials (membrane voltages) at 0.7 ms are hardly distinguishable. For the non-refined model, the agreement is worse (~20% on average). The results indicate that ‘inside’ the gray matter the BEM-FMM and SIMNIBS based modeling of the neuronal activation thresholds show robust agreement. On the other hand, axonal activation mechanisms may be influenced by the tissue heterogeneity (that causes charge accumulation on the conductivity boundaries) that may require more detailed analysis of the fields close to the gray-white matter boundaries [27]. Of course, the E-fields on the micro- and mesoscopic level are not fully characterized by the macroscopic model and how to merge the anatomical and histological information across multiple scales is a topic of further research.

### 4.4. Major limitations of the present direct solution

The present method has been tested on four multicore (32 to 56 cores) 2.6 GHz workstations. The minimum required RAM is approximately 64 Gigabytes. The FMM-LU factorization of the head-model specific system matrix requires approximately 40 min. The present method cannot run on a standard laptop.

As of today, neither BEM-FMM nor FMM LU are in position to handle macroscopic medium anisotropy. If the anisotropic part of the volume conductor model would be perfectly aligned and have the matching resolution, the error would be on the order of 10% with respect to the isotropic case as our computations for the healthy subject scanned at Max Planck Inst. for Human Cong. & Brain Sciences Leipzig, Germany show. However, the estimate of the anisotropy conductivity tensor based on the diffusion tensor imaging data is subject to its own challenges.

## 5. Conclusion

The direct TMS solver suggested in this study can determine global E-fields in modern high-resolution head models in approximately 5 seconds, which approaches the speed of single-pulse TMS motor mapping. There are no limitations on coil positions, coil types, coil deformations, ROI sizes, and ROI locations. For the standard head model, solver’s numerical accuracy is better than the accuracy of the widely used first-order FEM software when the isotropic head models are used. The FMM LU method can thus be employed to accelerate optimization problems in which a large number of coil configurations have to be simulated and a very large number of field values have to be computed. For example, approximately 24 hours would be necessary to accurately compute intracortical fields at 200,000 observation locations anywhere inside the brain volume for 1,000 TMS coil positions and 20 orientations per position.

The method could be readily expanded to other types of brain stimulation. Ref. [38] presents the standalone downloadable FMM LU code which replicates all data from Fig. 2, along with a short user’s manual. The computational platform is standard MATLAB running on Windows. The code accepts surface head meshes in STL format and is compatible with the coil models from the BEM-FMM package [10]. Ref. [38] also includes several movies recorded for examples from Fig. 2 and Fig. 3, respectively, and at the identical computational speed.

## Figures and Tables

**Table 1.** Field error values from Eq. (6) for the mid-surface between gray and white matter. The default Ernie model of the SimNIBS v3.2.6 FEM software with the default headreco segmentation and the default coil type/position is tested. Three solutions are considered: the default SimNIBS FEM solution, the iterative BEM-FMM solution, and the new BEM-FMM LU solution.

Error types and their percentage values	SimNIBS Ernie model	1:4 Uniform Refinement	1:16 Uniform Refinement
*Error*_*total*_ for entire midsurface/SimNIBS FEM	**27.1%**	-	-
*Error*_*total*_ for entire midsurface/iterative BEM-FMM	**14.7%**	1.7%	0.5%
*Error*_*total*_ for entire midsurface/BEM-FMM LU	**14.7%**	-	-
*Error*_*total*_ for 4 cm ROI under coil/SimNIBS FEM	**25.0%**	-	-
*Error*_*total*_ for 4 cm ROI under coil/iterative BEM-FMM	**3.3%**	0.4%	0.1%
*Error*_*total*_ for 4 cm ROI under coil/BEM-FMM LU	**3.3%**	-	-
			
*Error*_*mag*_ for entire midsurface/SimNIBS FEM	**17.7%**	-	-
*Error*_*mag*_ for entire midsurface/iterative BEM-FMM	**4.9%**	0.6%	0.2%
*Error*_*mag*_ for entire midsurface/BEM-FMM LU	**4.9%**	-	-
*Error*_*mag*_ for 4 cm ROI under coil/SimNIBS FEM	**5.7%**		
*Error*_*mag*_ for 4 cm ROI under coil/iterative BEM-FMM	**1.6%**	0.2%	0.06%
*Error*_*mag*_ for 4 cm ROI under coil/BEM-FMM LU	**1.6%**	-	-

**Table 2.** Field error values from Eq. (6) for the mid-surface between gray and white matter. The FMM-LU is tested with different values of FMM precision for (i) LU matrix decomposition; and (ii) field computations. Four Connectome models are tested along with the default Ernie model of the SimNIBS FEM software. The superscript denotes standard deviation.

Error type and percentage values	SimNIBS Ernie model	Connectome models, averaged
FMM LU precision: 1e-4: Field FMM precision: 1e-4		
*Error*_*total*_ for entire midsurface/BEM-FMM LU vs. iterative sol.	0.10%	0.11%^0.02%^
*Error*_*total*_ for 4 cm ROI under coil/BEM-FMM LU vs. iterative sol.	0.03%	0.03%^0.003%^
*Error*_*mag*_ for entire midsurface/BEM-FMM LU vs. iterative sol.	0.08%	0.08%^0.01%^
*Error*_*mag*_ for 4 cm ROI under coil/BEM-FMM LU vs. iterative sol.	0.03%	0.02%^0.005%^
FMM LU precision: 1e-4: Field FMM precision: 1e-1		
*Error*_*total*_ for entire midsurface/BEM-FMM LU vs. iterative sol.	0.37%	0.38%^0.02%^
*Error*_*total*_ for 4 cm ROI under coil/BEM-FMM LU vs. iterative sol.	0.15%	0.16%^0.03%^
*Error*_*mag*_ for entire midsurface/BEM-FMM LU vs. iterative sol.	0.28%	0.27%^0.02%^
*Error*_*mag*_ for 4 cm ROI under coil/BEM-FMM LU vs. iterative sol.	0.15%	0.14%^0.01%^
FMM LU precision: 1e-3: Field FMM precision: 1 e-1		
*Error*_*total*_ for entire midsurface/BEM-FMM LU vs. iterative sol.	1.35%	1.46%^0.29%^
*Error*_*total*_ for 4 cm ROI under coil/BEM-FMM LU vs. iterative sol.	0.40%	0.42%^0.16%^
*Error*_*mag*_ for entire midsurface/BEM-FMM LU vs. iterative sol.	1.05%	1.04%^0.19%^
*Error*_*mag*_ for 4 cm ROI under coil/BEM-FMM LU vs. iterative sol.	0.39%	0.37%^0.18%^

## Data Availability

The standalone executable computer code that supports the findings of this study along with six short real-time movies is available from repository of Ref. [38]: *TMS FMM-LU March. 2023: Source code in MATLAB and videos*: DropBox: https://www.dropbox.com/sh/ztra43jfj8afh0z/AAAG8mdqyjkQR9UCWYrJQ14Ha?dl=0
